# Efficient Coverage Path Planning for Mobile Disinfecting Robots Using Graph-Based Representation of Environment

**DOI:** 10.3389/frobt.2021.624333

**Published:** 2021-03-15

**Authors:** B. Nasirian, M. Mehrandezh, F. Janabi-Sharifi

**Affiliations:** ^1^ Faculty of Engineering and Applied Science, University of Regina, Regina, SK, Canada; ^2^ Robotics, Mechatronics and Automation Laboratory (RMAL), Department of Mechanical and Industrial Engineering, Ryerson University, Toronto, ON, Canada

**Keywords:** coverage path planning, disinfection, optimization, deep reinforcement learning, autonomous mobile robots

## Abstract

The effective disinfection of hospitals is paramount in lowering the COVID-19 transmission risk to both patients and medical personnel. Autonomous mobile robots can perform the surface disinfection task in a timely and cost-effective manner, while preventing the direct contact of disinfecting agents with humans. This paper proposes an end-to-end coverage path planning technique that generates a continuous and uninterrupted collision-free path for a mobile robot to cover an area of interest. The aim of this work is to decrease the disinfection task completion time and cost by finding an optimal coverage path using a new graph-based representation of the environment. The results are compared with other existing state-of-the-art coverage path planning approaches. It is shown that the proposed approach generates a path with shorter total travelled distance (fewer number of overlaps) and smaller number of turns.

## Introduction

Surfaces contaminated with COVID-19 pathogens in hospitals introduce significant risk to the safety of medical personnel and patients. Disinfection routines are among critical measures that hospitals are taking to minimize the spread of COVID-19. To reduce the workload of the hospitals’ cleaning teams and to avoid the direct contact of disinfecting agents such as chemicals or UV-C disinfectants with human body, autonomous mobile robots would provide a favorable solution. The autonomous robots can potentially perform the disinfection task more precisely and in a timely and cost-effective fashion. Central to robotic disinfection routines is the coverage path planning.

Coverage Path Planning (CPP) will lead to an improvement in the efficiency of operations in terms of cost, time, and job quality. It is defined as: generating a continuous and un-interrupted path that covers an area of interest, while avoiding obstacles (Galceran and Carreras, 2013). The efficiency of a CPP algorithm is usually determined by the total coverage ratio, completion time, total travelled path length, and the number of turns (Khan et al., 2017).

Some CPP works cited in the literature are based on heuristics or randomized approaches, where the coverage path is determined based on a set of simple behaviors (e.g., Mackenzie and Balch, 1993) or randomized search through the environment (e.g., Palacin et al., 2005). These methods, however, do not guarantee a complete coverage of the free space (Choset, 2001) while coverage completeness is essential to guarantee that all COVID-19 pathogens are killed during the robotic disinfection tasks.

Complete CPP methods decomposed the free space into smaller regions (cells) in which optimal path planning could be simply formulated (Choset, 2001). A complete coverage was achieved by ensuring that the robot visited all cells in the decomposed environment at least once (Choset, 2001). Among the decomposition methods cited in the literature, an exact environment decomposition method would stand out specially in environments with un-even-shape boundaries and in presence of concave-shape obstacles since the re-union of the cells under this decomposition method would fully represent the free space (Cabreira et al., 2019). In the pertinent literature, three topics are given special attention: 1) the environment decomposition techniques, 2) the optimal coverage path in each cell generated via environment decomposition, and 3) the optimal coverage sequences (Cabreira et al., 2019). In this paper we formulate a complete coverage path planning in the environment that leads to a minimal travelled distance (cost).

The operational environment would generally consist of obstacles, free space, and the mobile robot. In the Boustrophedon decomposition, as the most commonly-used exact cellular decomposition approach, the free space was divided into smaller regions (cells) by sweeping a line through the whole target area in one direction (Choset and Pignon, 1998; Choset et al., 2000). In order to traverse from one cell to another, the robot might need to transit through a part of a third cell. This causes overlaps, thus, extra travelled distance. This is mainly due to the fact that no mechanism has been considered for transition from one cell to another in present exact cellular decomposition methods. To avoid the unnecessary cost associated with this transition, a modified version of the Boustrophedon-based decomposition has been proposed by us. Three transition cells have been added to the decomposed environment at each critical point. This allows the robot to either expand or shrink the original cells around the critical point to avoid the overlaps of inter-cell traversals and cell coverage paths (further explained in Section *Modified Graph Considering the Modified Environment Decomposition (to Avoid Overlaps)*).

Under classical CPP approaches (e.g., Choset and Pignon, 1998; Choset et al., 2000), an adjacency graph was built based on the topology of the decomposition, where the nodes of the graph represented the cells and the edges of the graph connected the nodes with adjacent corresponding cells in the decomposed environment. The problem of finding the optimal coverage sequence was equivalent to finding the shortest path within the adjacency graph that visits each node (cell) at least once, which was equivalent to the Traveling Salesman Problem. The problem of finding the optimal path over the adjacency graph is an NP-complete problem. A depth-first graph search algorithm was proposed to find an exhaustive walk through the adjacency graph. However, the depth-first search solution was not optimal, was computationally expensive, and required huge memory storage for problems with a large adjacency graph (i.e., environments with a large number of cells). Later works on CPP (e.g., Jimenez et al., 2007; Hameed et al., 2013; Tung and Liu, 2019) utilized Genetic Algorithm (GA) optimization techniques to find an efficient coverage sequence over larger adjacency graphs in a shorter computational time and with less required memory space. However, in the coverage path found using the adjacency graphs the robot usually needs to traverse through the middle of the cells to transit from one cell to another, which results in overlaps (extra travelled distance). The shortest path that visits all nodes of the adjacency graph is not necessarily equivalent to the shortest path travelled by robot since the graph does not consider the overlaps of the transition paths with coverage back-and-forth straight-line motions in the cell.

Another approach to find the traversal sequences was to create a Reeb graph of the environment, where the nodes denoted the critical points, and the edges represented the cells (Mannadiar and Rekleitis, 2010). In order to find the most efficient coverage sequence, the Chinese Postman Problem was solved over the graph, that was to find the shortest tour that traversed over every edge at least once (Mannadiar and Rekleitis, 2010). In (Mannadiar and Rekleitis, 2010; Xu et al., 2011; Xu et al., 2014), the Reeb graph was modified to an Eulerian graph by duplicating selected edges of the graph. The edge duplication referred to a situation, where the cell corresponding to the duplicated edge in graph was divided into two different cells, which further leaded to extra turns to make in the middle of the original cell. Since performing a turn in the path takes more time and energy than that in a straight-line motion, an efficient coverage path should be generated such that the total number of turns is minimized and consequently the total operational time and cost of the CPP is decreased (Galceran and Carreras, 2013).

In this work, a CPP approach is proposed that works based on a new graph representation of environment. In order to avoid the costly turns in the middle of the cells, which is the case in (Mannadiar and Rekleitis, 2010; Xu et al., 2011; Xu et al., 2014), and transition path overlaps through the middle of the cells, which is the case in (Jimenez et al., 2007; Hameed et al., 2013; Tung and Liu, 2019), two different possible actions have been considered for the robot in a cell: 1) back-and-forth straight-line motion with turns at the end of the lines for covering the cell, and 2) environment/obstacle contour-following motion to adjust the robot position for starting the cell coverage. At those cells, which had their corresponding Reeb graph edge duplicated in (Mannadiar and Rekleitis, 2010; Xu et al., 2011; Xu et al., 2014), the robot will have the option to follow some parts of the cell’s contour in the first traversal, and then cover the cell by back-and-forth straight-line motion in the second traversal without any overlaps with the covered parts of the cell’s contour in first traversal. This would lead to a new form of graph in which the Eulerian cycle/path needs to be determined leading to a minimum travelled distance (overlaps). The number of turns would be also less than that cited in literature, (e.g., Xu et al., 2014), since the turns in the middle of the cells are eliminated (this will be further explained in Section *Modified Graph Considering Contour-Following Motion*).

Another contribution of this research is that it evaluates the inter-cell traversals at a low-level as well. The travelled distance depends on both current-cell-coverage end point and next-cell-coverage start point. Most of the previous works on CPP (e.g., Choset and Pignon, 1998; Jimenez et al., 2007; Xu et al., 2014) did not consider the current position of the robot in the current cell to choose the next cell in the coverage sequence. In (Chen et al., 2019), a corner model was utilized to find the shortest path between the current cell and the next cell which would not necessarily lead to an optimal path that robot can take. Their technique did not choose the corner that cell coverage should be started from, hence, the corner information was not included in their graph representation of the environment. In (Hameed et al., 2013; Tung and Liu, 2019), entrance and exit points have been considered for each cell which resulted in multiple inter-cell paths for each cell. In this work, four corners have been considered at each cell as the candidates for the cell coverage start and/or end points. Contrary to the case in a Reeb graph, where the nodes represent the critical points, in the proposed graph by us, the nodes represent the cell corner points at the critical points. Some extra edges get added to the graph, which facilitate inter-cell traversal paths at each critical point. To perform a complete coverage, some edges are required to be traversed (cell coverage edges), while some other will remain optional (i.e., contour-following, and inter-cell traversal edges) (for further explanation see Section *Modified Graph Considering Cell Coverage Start and End Points*).

The problem of finding the efficient cell coverage sequence can be solved by finding a path over the proposed graph in which a required subset of the edges is needed to be traversed with minimal cost. In this work, the cell coverage sequence optimization problem has been considered as a Markov Decision Process (MDP), and the efficient sequence within the graph has been found using a double Deep Q Network (DQN) approach. Double DQN is a Deep Reinforcement Learning (DRL) method which utilizes two identical deep neural networks to estimate Q-values where each of the networks is used to update the other. The efficient coverage sequence of the cells is equivalent to the optimal policy found via the double DQN. In addition to finding shortest travelled distance, the path generated through this method is robust to changes in the start and/or end positions of the disinfection task and works for coverage of any arbitrary subset of the cells in the target space (further explained in Section *Coverage Optimization Over the Proposed Graph*).

The aim of this work is to decrease the disinfection task cost by adopting a new graph-based representation of the environment. More specifically, the contributions are:

- A new graph representation of the environment has been proposed based on the following modifications which facilitate finding an efficient coverage path for disinfection task.
  1) Proposing a modified version of the Boustrophedon environment decomposition with three transition cells added at each critical point to allow the original cells expansion or shrinkage;
  2) Programming two different actions for the robot in a cell: 1) back-and-forth straight-line motion, and 2) contour-following motion to adjust the robot position for starting the cell coverage with no extra travelled distance (overlaps); and
  3) Considering the corners of the cells (as the candidates for the cell coverage start and/or end points) as graph nodes to minimize the inter-cell traversal path overlaps at each critical point.
- The optimization problem over the proposed graph has been solved using a Double DQN technique which trains a model over the environment to find an efficient coverage path for any start and/or end positions of the disinfection task and any arbitrary subset of the cells in the environment.

The results of the proposed approach are compared with other complete CPP approaches cited in literature for indoor environments (see Section *Results and Discussion*). It is shown that the proposed approach outperforms the previous techniques in terms of the total travelled distance. In addition, the total number of turns are reduced in comparison with the work in (Xu et al., 2014). Also, the proposed method is robust to changes in the start and/or end positions of the robot used for the disinfection task, and that it generates coverage path for any arbitrary set of the cells in the decomposed environment. This will reduce the overall cost of repetitive disinfection tasks in large hospitals drastically.

## Methodology

In order to decrease the travelled distance and the number of turns in the hospital disinfection task, a complete CPP based on a new graph representation of the decomposed environment has been proposed. In this section the steps and approaches of environment decomposition, constructing the graph, and solving the optimization problem over the graph are described.

### Environment Decomposition

**FIGURE 1:**
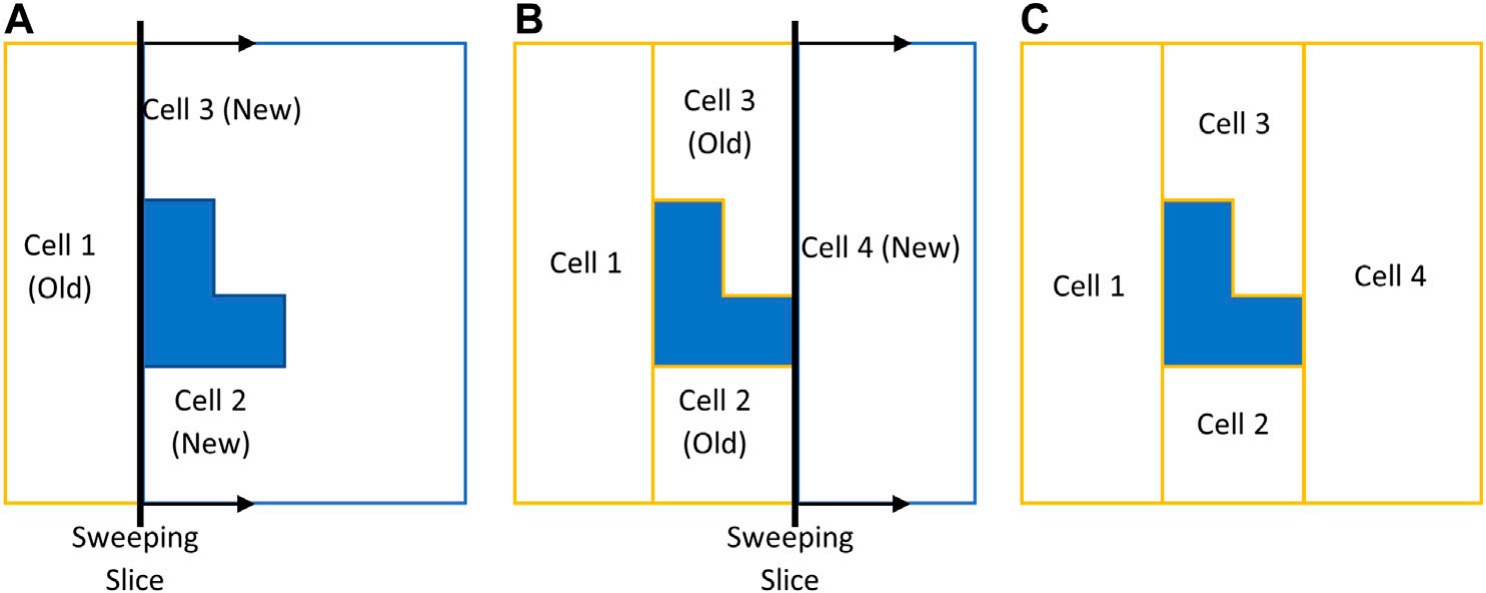
Boustrophedon decomposition technique with a slice sweeping from left to right. **(A)** Slice connectivity changes from one to two which results in two new cells. **(B)** Slice connectivity changes from two to one which results in one new cell. **(C)** Decomposed environment.

CPP in environments comprised of non-convex boundaries and obstacles renders itself as a complex problem. A commonly-used technique in most CPP approaches is to decompose the environment using exact cellular decomposition into smaller regions (cells) in which optimal path planning can be formulated (Choset, 2001). Boustrophedon decomposition is one of the most commonly-used exact cellular decomposition methods for CPP problem over planar environments. The Boustrophedon decomposition assumes the environment boundaries are polygonal and known a priori. In this method, a line segment (called a slice) is swept through the whole target area in one direction to determine the critical points. Critical points are the vertices of the environment boundaries where the sweeping slice connectivity changes (Choset and Pignon, 1998). One or two new cells are formed whenever the slice arrives at a critical point, as shown in Figure 1. The decomposition can be done in different directions (i.e., different slice sweeping directions) resulting in different cell decomposition configurations. One can also optimize the coverage by finding the best decomposition direction over a particular environment which leads to a cost-efficient coverage path (Oksanen and Visala, 2009).

After decomposing the environment into cells, the optimal coverage path inside each cell can be determined separately by minimizing a coverage cost function. The direction of the back-and-forth straight-line motions can be determined in a way that the total number of turns is minimized and consequently the total operational time of the CPP is decreased (Galceran and Carreras, 2013).

### Graph Representation of Decomposed Environments

In order to find an efficient sequence in traversing all the cells in a decomposed environment, a commonly-used approach is to build a graph that captures the topology of the cells. The problem of finding the minimum cost coverage sequence is then equivalent to finding the shortest path over this graph. In this work, we have proposed a new graph representation of the environment which leads to a more efficient coverage path. More details are provided in the following sub-sections.

#### Mobile Robot

The proposed CPP technique in this work focuses on finding the efficient sequence of cells coverage and the inter-cell paths connecting the cells using a graph representation of the environment. Similar to some other suggested CPP-based techniques cited in the literature (e.g., Choset and Pignon, 1998; Mannadiar and Rekleitis, 2010; Hameed et al., 2013; Chen et al., 2019), the proposed CPP technique in general is not limited to a particular robot and is implementable on most of the mobile disinfecting robots available in the market. However, some assumptions have been made on the mobile robot shape, disinfection system, and the drive mechanism when in designing the proposed graph representation and the coverage path over the environment.

The mobile robot is assumed to have a disk shape (i.e., the robot is presented as a circle that circumscribes the robot footprint entirely). Also, we assume that the robot is equipped with a UV-C disinfection system. In order to avoid collision with environment boundaries, the diameter of the circle circumscribing the robot (L) should be known while generating the proposed graph and the coverage path (equivalent to constructing the configuration space). Furthermore, disinfection diameter (D) denotes the diameter of the area that the robot can disinfect using its onboard probes, e.g., the maximum range of the onboard UV-C lamp array can be considered as the disinfection radius. Disinfection diameter (D) is always greater than or equal to the diameter of the largest circle that circumscribes the entire robot (L). It is also assumed that the disinfecting robot is of a differential-drive type, which is capable of turning on the spot. Therefore, the robot does not need any extra space to perform turns at the end of the straight-lines in the planned path.

In order to disinfect things such as the beds, walls, shelves, and other equipment in the hospitals, the disinfection coverage diameter (W) is assumed to be always smaller than or equal to the disinfection diameter (D). Please note that, in the coverage path, the distance between the stripes and the distance of the robot’s center point to the boundaries of the obstacles are equal to W and W/2, respectively. This means that the free space will get disinfected based on the disinfection coverage diameter (W), while a depth of (D/2-W/2) of the walls and obstacles are being disinfected (note that D ≥ W ≥ L). All the items within the range (i.e., at the D/2 distance from the center of the robot) along the path will be disinfected. We also assume that, in addition to vertical UV-C emitters, there are circumferential emitters to disinfect blind spots immediately under and/or surrounding the robot. If there are goods covered by other objects, they will likely be missed due to the nature of radiation-based disinfection. Since W is always greater than or equal to L, it is guaranteed that the robot will not collide with the environment boundaries (equivalent to constructing an automaton representation of a point robot within its configuration space). Robot diameter (L) and disinfection diameter (D) depend on the mobile robot used for the disinfection task while the disinfection coverage diameter (W) can be selected by a user based on the required obstacles disinfection depth (D/2-W/2). The disinfection coverage diameter (W) is an input to the proposed algorithm in this work, and it can be adjusted by the user. In all figures and results presented in this paper, the default value of W is assumed to be equal to 1 m. The algorithms would allow different W values, however.

#### Reeb Graph

**FIGURE 2:**
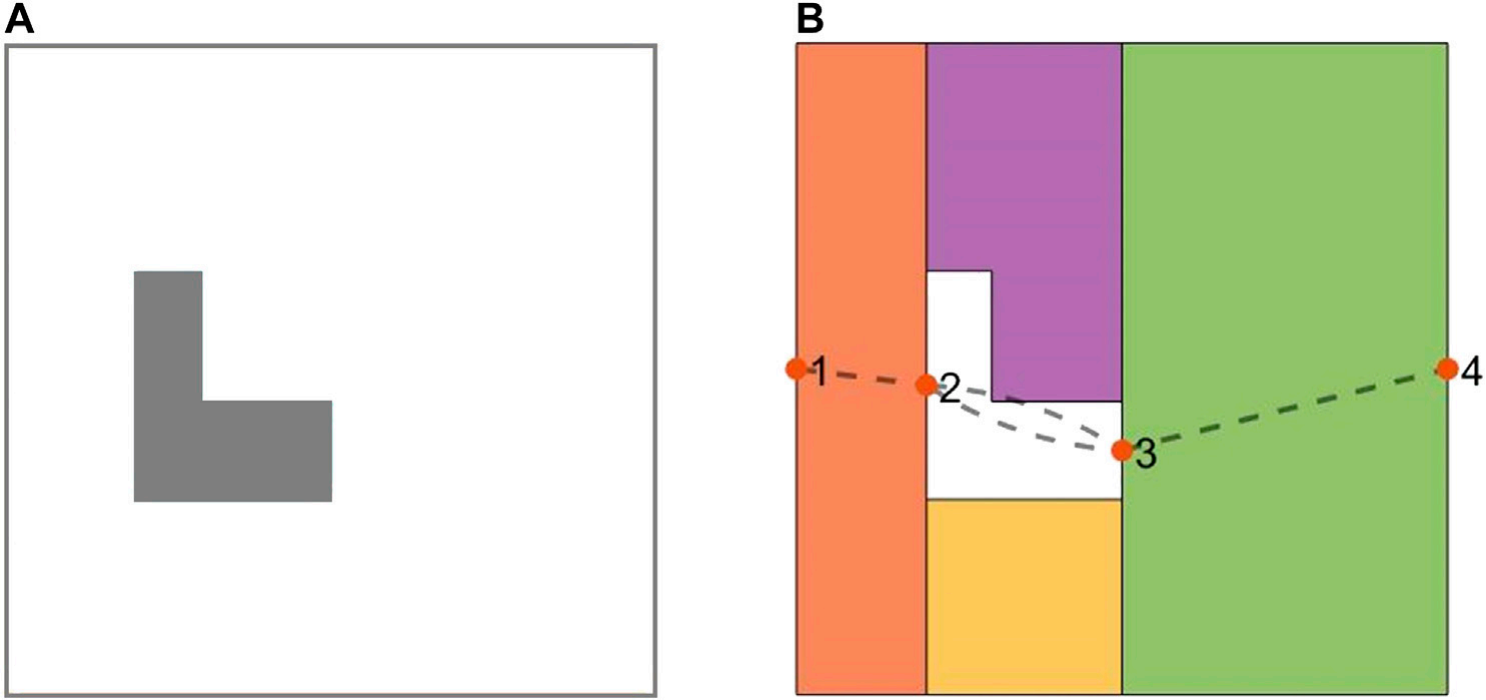
**(A)** The planar map of a simple indoor environment in presence of a convex obstacle. **(B)** The Boustrophedon decomposition and Reeb graph representation of the environment. The numbered solid points represent the nodes, and the dashed lines denote the edges in the Reeb graph.

One of the approaches that has been utilized in the literature is based on generating a Reeb graph representation of the environment and to solve the Chinese Postman Problem over that graph (Mannadiar and Rekleitis, 2010). Under this, the nodes denote the critical points, and the edges represent the cells connecting two neighboring critical points. Figure 2 illustrates the Boustrophedon decomposition and Reeb graph representation of a simple environment in presence of a convex-shape obstacle. In Figure 2, the Reeb graph contains four nodes (critical points) and four edges (cells).

In order to find the best coverage sequence of the cells, one needs to solve the Chinese Postman Problem on the graph, which translates to: finding the shortest tour that traverses every edge on the Reeb graph at least once. If the Reeb graph of the environment is an Eulerian graph, all its Euler tours will be solutions to the Chinese Postman Problem (Mannadiar and Rekleitis, 2010). For non-Eulerian Reeb graphs, a standard approach to solve the Chinese Postman Problem is to modify the graph to an Eulerian one by duplicating selected edges in the graph. The challenge is to choose duplicated edges such that the total cost (the sum of the individual costs of all the edges) of the Euler tour be minimized (Mannadiar and Rekleitis, 2010). Different strategies such as linear programming and matching theory algorithms can be utilized to determine which edges to duplicate (Edmonds and Johnson, 1973).

However, the generated paths in (Mannadiar and Rekleitis, 2010; Xu et al., 2011; Xu et al., 2014) include a high number of turns in the middle of the environment because of dividing the cells with duplicated edges into two parts. In addition, the travelled distance can decrease by applying some modifications to the Reeb graph. Our proposed approach to resolve these short comings by modifying the Reeb graph is described in the following sections.

#### Modified Graph Considering Cell Coverage Start and End Points

**FIGURE 3:**
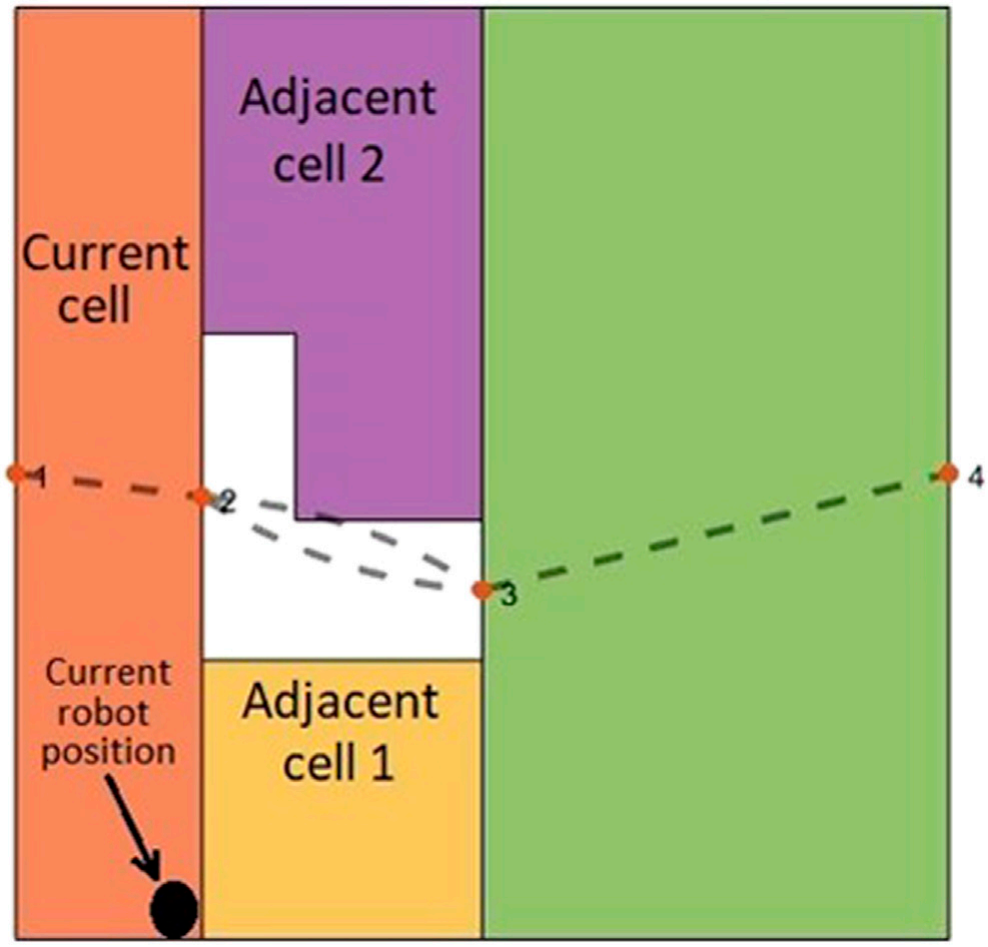
The position of the mobile robot along the common boundary is an important factor in choosing one of the adjacent cells as the next cell in the coverage sequence.

Most of the previous works on CPP did not consider the position of the mobile robot in the current cell to choose the next cell in the coverage sequence. As shown in Figure 3, if the mobile robot is at the common boundary of the current cell (already covered cell) and two adjacent cells (not covered yet), the position of the mobile robot along the common boundary would be an important factor to account for when choosing the next cell in the coverage sequence. For example, when the robot is at the position shown in Figure 3, the adjacent cell one would be a better candidate, than adjacent cell 2, to be the next cell in the coverage sequence. In (Chen et al., 2019), the shortest path between the current cell and the next cell is obtained by using a corner model. However, the shortest path between them is not always the best path. Graph-search approaches under CPP do not consider this point because graphs, particularly Reeb graphs, do not contain any information about the position of the robot at the critical points.

**FIGURE 4:**
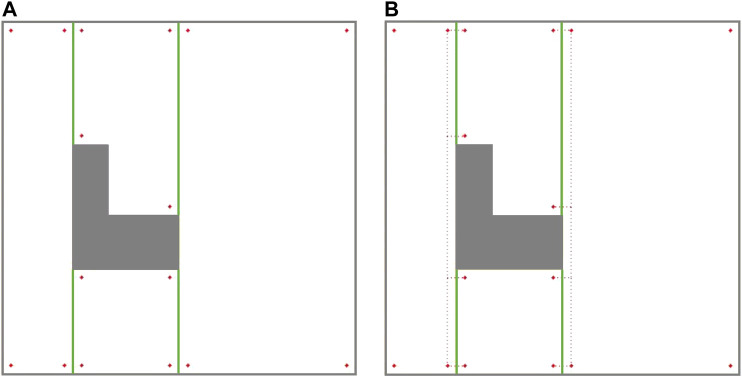
**(A)** The candidate cell coverage with start and end points at each cell. **(B)** The inter-cell transition options for mobile robot at each corner of the cells. The dashed red lines show the inter-cell transition paths.

The Reeb graph representation of the environment needs to be modified to include the information on position of the robot at the critical points. As illustrated in Figure 4A, four corners are being considered for every cell as the potential cell coverage start and end points. There are two critical points at two sides of a cell (left critical point and right critical point). In each cell, two corners are located on the right side of the left critical point (left-top and left-bottom), and two corners are located on the left side of the right critical point (right-top and right-bottom).

As it can be seen in Figure 4A, corners have an offset of W/2 from critical points horizontal position and environment boundaries, with W being the robot coverage diameter. This offset ensures that: 1) the robot does not collide with the environment boundaries (equivalent to constructing an automaton representation of a point robot within its configuration space) of the environment and 2) the covered areas at the common boundary of two adjacent cells do not overlap.

Figure 4B shows that the robot located at each corner of the current cell will have four optional paths to traverse to reach one of the four adjacent-cell corners. One should note that corners at the most left and most right critical points of the environment are exceptions. These optional paths can be added to the Reeb graph as some extra edges, which facilitate the inter-cell traversal at each critical point.

**FIGURE 5:**
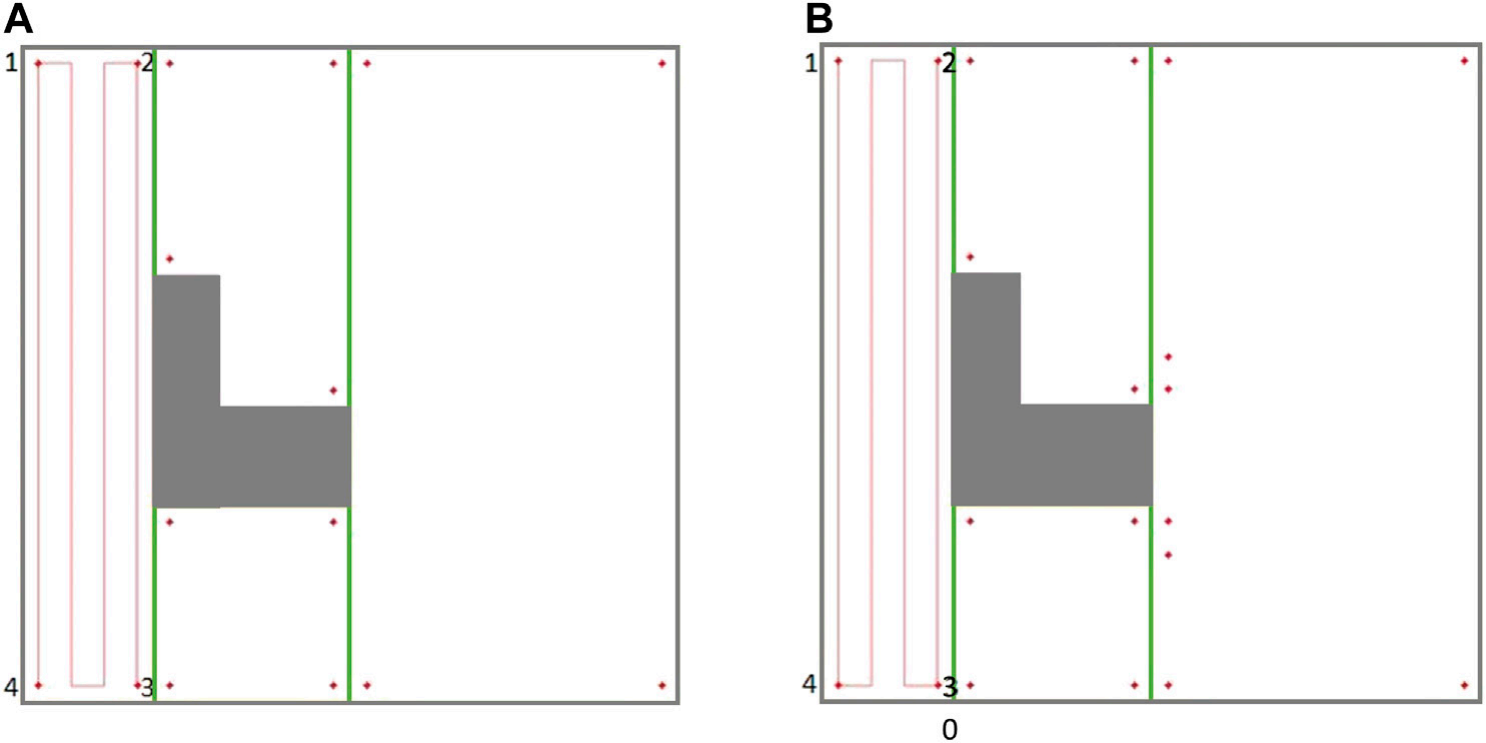
The coverage paths of a cell starting from different corners. **(A)** Cell coverage started from corner 4 ending at corner 3, and vice versa. **(B)** Cell coverage started from corner 1 ending at corner 2, and vice versa.

In the proposed modified graph, nodes denote the cell corners (not the critical points), and some optional edges are added to the graph representing the paths between the corners. In addition to the nodes, the Reeb graph edges need to be modified as well. Traversing each edge of the Reeb graph is equivalent to the corresponding cell coverage. However, as it is shown in Figure 5, the cell coverage can be done under two different options. Under the first cell-coverage option, if the robot starts the cell coverage from the left-bottom corner (corner 4) of the current cell, it will finish the coverage in one of the two right corners depending on the cell width and disinfection coverage diameter. In Figure 5A, the cell coverage finishes at the right-bottom corner (corner 3). The coverage path is undirected, so the coverage can start from corner three and end at corner 4. Under the second cell-coverage option, if the robot starts the cell coverage from the left-top corner (corner 1) of the same cell, the end corner on the right side (right-top corner or corner two in Figure 5B) would be different than the end corner in the first cell coverage option. Therefore, each edge of the Reeb graph should be replaced with a pair of coverage edges, where traversing only one of these edges will suffice for cell coverage.

**FIGURE 6:**
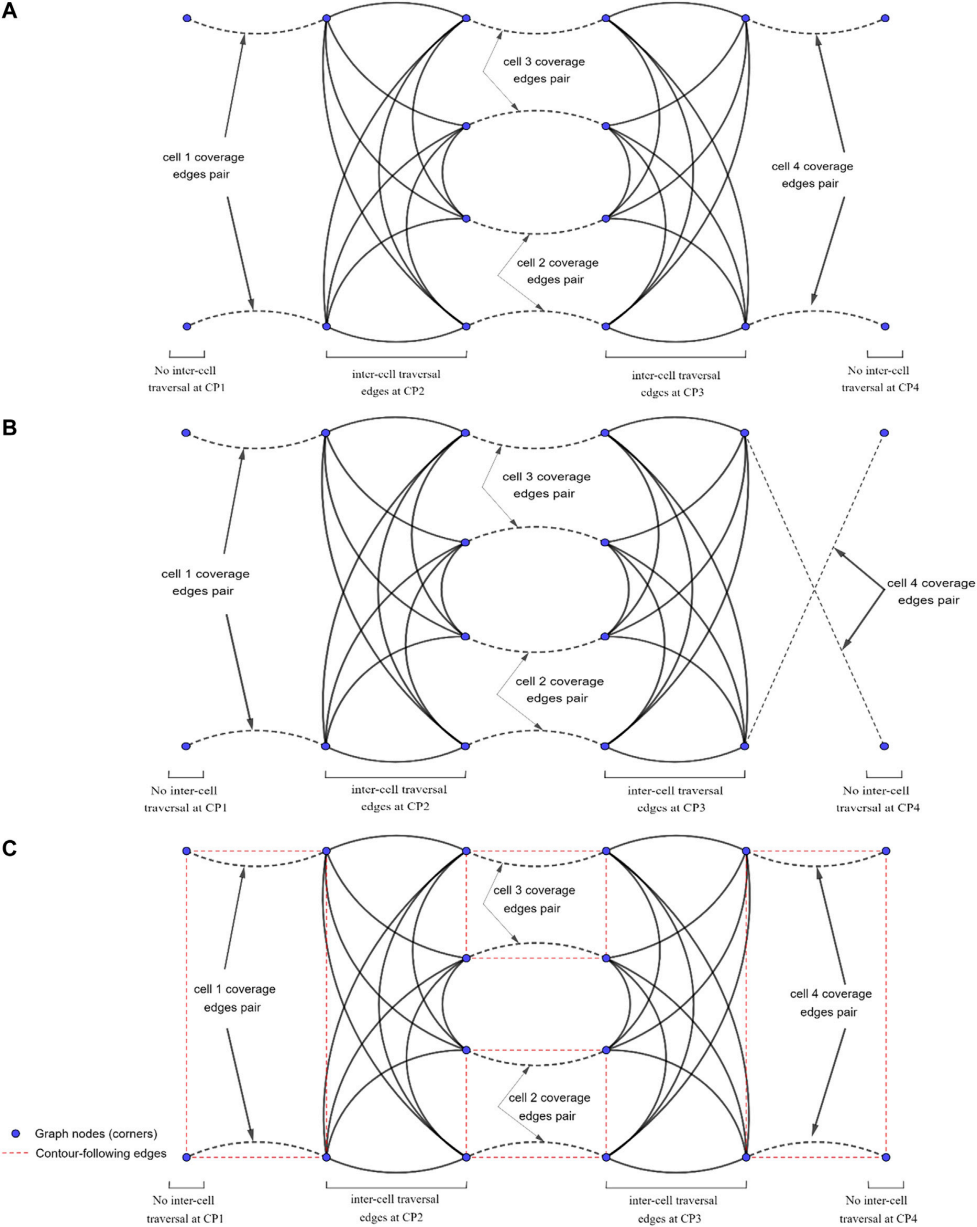
The graph representation of the simple environment of Figure 2 with coverage edges pairs and inter-cell traversal edges at critical points (CPs). **(A)** The quotient in dividing cell 4’s width by the coverage diameter (W) is even numbered. **(B)** The quotient in dividing cell 4’s width by the coverage diameter (W) is odd numbered. **(C)** Contour-following edges are added to the graph.

This would lead to a new graph-search problem in which some edges are required to be traversed (one of the two cell coverage edges at each cell), while some other edges will remain optional (inter-cell traversal edges in Figure 4B). Closest problem cited in the literature to this setup would be the Rural Chinese Postman Problem, where a subset of the edges from the graph are required to be traversed at a minimal cost. Since this required subset does not form a weakly-connected network, the Rural Chinese Postman Problem would constitute an NP-complete problem (Pearn and Wu, 1995). As opposed to that in the original Rural Chinese Postman Problem, there is a pair of coverage edges associated to each cell in the graph, and only one of these coverage edges is required to be traversed in our case. This means that there are no edges that are required to be traversed; however, there are pairs of edges that remain essential to be traversed. When one of the edges in a pair is traversed, that pair is considered to be complete. The graph representation of the simple environment seen in Figure 2, with coverage edge pairs and inter-cell traversal edges at each critical point (CP) is shown in Figure 6. The environment boundaries and cell decomposition have been removed from that in Figure 6 to illustrate that the cell coverage sequence problem can be solved as a solely graph-search problem indeed. In Figure 6A, all the quotients in dividing cells’ widths based on the coverage diameter (W) in this environment are even numbered. Therefore, the coverage edges’ end points are adjacent to their start points. Figure 6B shows a case where the quotient in dividing cell 4’s width by the coverage diameter (W) is odd numbered. As it can be seen in Figure 6B, the coverage edges in cell four start and end corners are not adjacent. It should be noted that the proposed graph representation of environment is undirected; therefore, both coverage and inter-cell traversal edges do not have a direction.

#### Modified Graph Considering Contour-Following Motion

**FIGURE 7:**
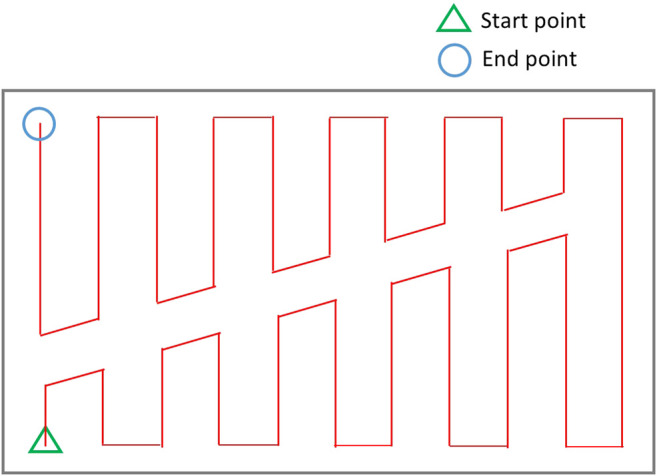
Coverage of a single cell in (Mannadiar and Rekleitis, 2010).

As it can be seen in Figure 6A, in order to find a route over the graph which traverses all coverage edge pairs, some of the coverage edges would be required to be duplicated or at some cases both coverage edges in the same pair have to be traversed. In (Mannadiar and Rekleitis, 2010; Xu et al., 2011; Xu et al., 2014), in order to avoid covering the cells with duplicated edges twice, the graph-search algorithm was modified to cover the top (or bottom) part of the cell in the first traversal and the bottom (or top) part of the cell in the second traversal. However, as Figure 7 shows, the generated path includes a high number of turns right in the middle of the environment because of splitting cells. One should note that turns are more costly, so they have to be avoided. The travelled distance is also increased under this algorithm because of the extra distance travelled to make those turns.

Instead of splitting the cells into two, we have defined a contour-following option for the mobile robot. It allows the robot to adjust its position in a cell, or cross a cell, without traversing cell coverage edges. This will result in a reduced number of turns and travelled distance. Two different actions are considered for the robot inside each cell: 1) back-and-forth straight-line motion with turns at the end of the lines (cell coverage edges pair), and 2) contour-following motion to adjust the robot position for starting the cell coverage (contour-following edges).

**FIGURE 8:**
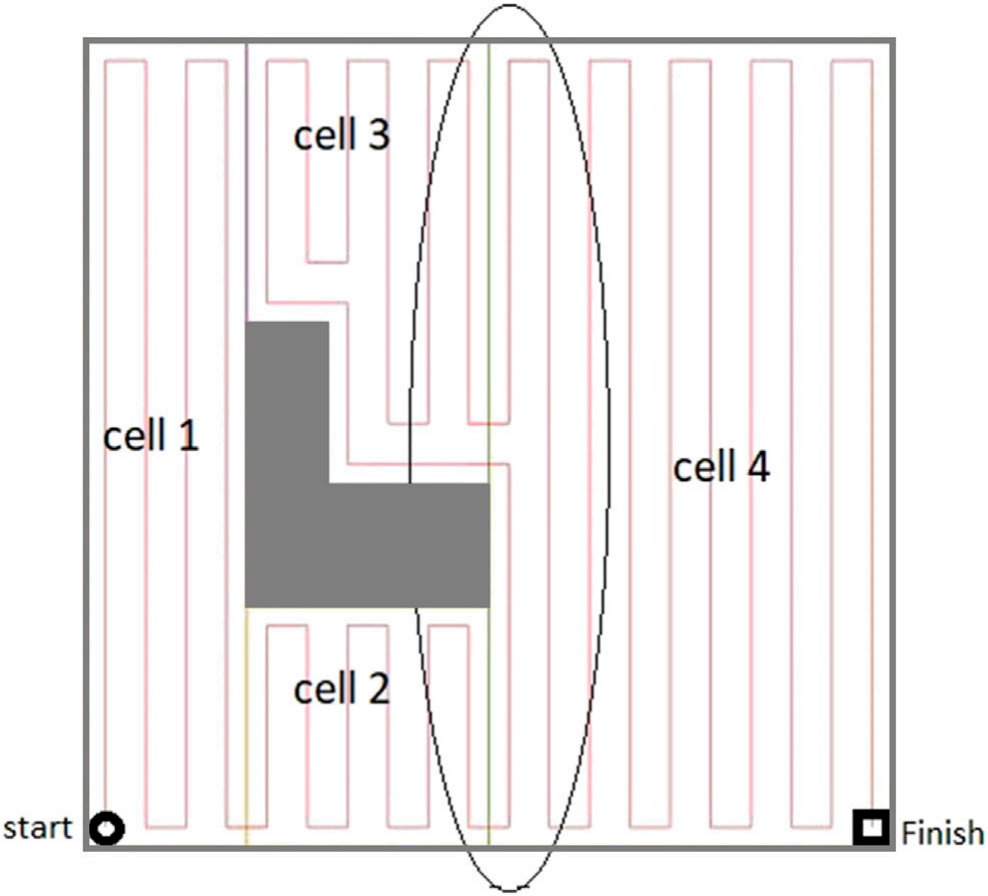
The optimal coverage path for the simple environment of Figure 2. The ellipse shows the first stripe of cell four which has been covered apart from the rest of the cell area.

In this approach, instead of duplicating the coverage edges or traversing both cell coverage edges in the same pair, the mobile robot follows some parts of the cell’s contour in the first traversal to get to a favorable corner node to start the cell coverage from. Then, it covers the rest of the cell by back-and-forth straight-line motions in the second traversal. An optimal coverage path over the environment in Figure 2 has been represented in Figure 8. A contour-following motion has been performed by the mobile robot in cell 3. As it can be seen in Figure 8, there would be no overlaps between the back-and-forth straight-line motions and the path taken by robot to adjust its position for starting cell three coverage which was not the case in CPP techniques (Jimenez et al., 2007; Hameed et al., 2013; Tung and Liu, 2019) that utilized adjacency graph to find the coverage sequence.

There are four possible contour-following paths at each cell connecting the adjacent cell corners to each other. The contour-following paths have been added to the graph (see Figure 6C). In CPP problem, traversing of the contour-following edges is not required.

#### Modified Graph Considering the Modified Environment Decomposition (to Avoid Overlaps)

**FIGURE 9:**
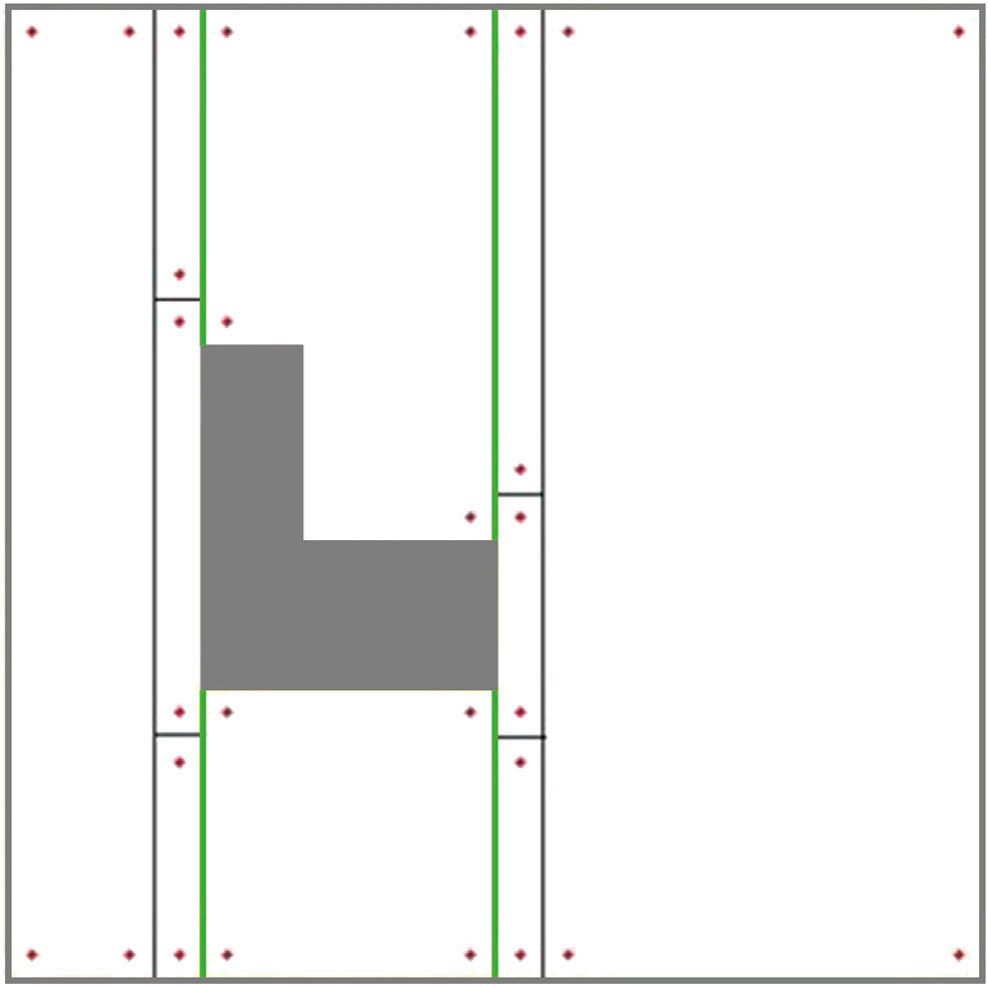
The modified cellular decomposition with transition cells. Each transition cell has only two corners.

**FIGURE 10:**
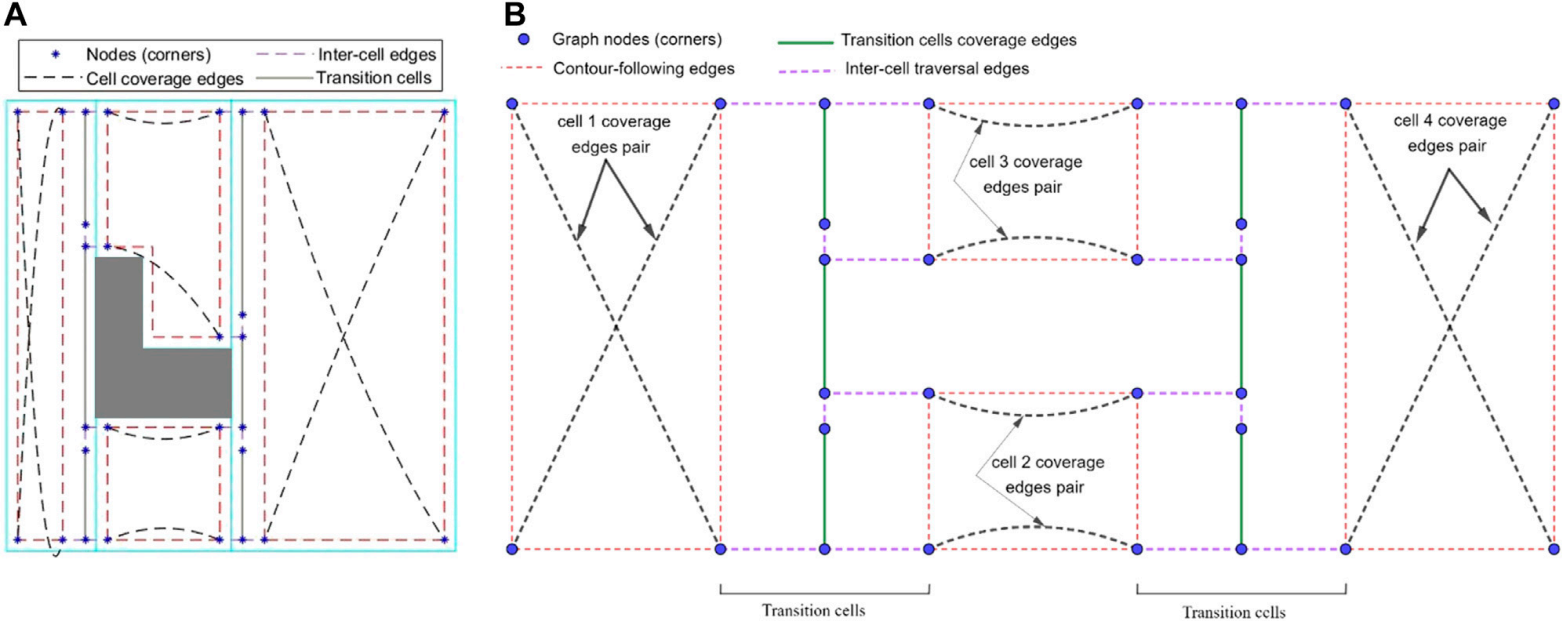
The graph representation of the simple environment of Figure 2, which can be utilized to find an efficient cell coverage sequence. **(A)** The environment boundaries are shown to present how the graph is constructed. **(B)** The environment boundaries are removed to show that the coverage sequence problem can be solved as a solely graph problem.

The ellipse shown in Figure 8 represents a part of the optimal path over the environment, where the most left stripe of cell 4 has been covered separately while the robot was traversing from cell two to cell three and from cell two to cell four. Cell four coverage has started from the second stripe of the cell. The graph does not contain enough information to consider skipping the first stripe. When the robot is traversing a cell coverage edge, it is covering the whole cell. As a result, there would be a coverage overlap in the first stripe of the cell four. This overlap increases the travelled distance and consequently the cost of operation. To avoid this, the graph has been modified based on a more flexible environment decomposition method. Three transition cells are added to the cellular decomposition of the environment at each critical point. This allows the robot to expand or shrink the cells around the critical points to avoid the overlaps of inter-cell traversals and cell coverage paths. Figure 9 illustrates the transition cells and their associated corners which are added to the graph nodes. The transition cells have a width of W (coverage diameter) and include two corners (top and bottom). The modified graph of the simple environment of Figure 2 has been shown in Figure 10. In Figure 10A, the graph is represented over the environment. The environment boundaries are removed in Figure 10B to illustrate that the coverage sequence problem can be considered as a solely graph-search problem. Those edges of the graph that are equivalent to coverage of transition cells should be added to the list of required edges to be traversed for complete coverage. All other added inter-cell traversal edges at the end of the transition cells are optional.

### Coverage Optimization Over the Proposed Graph

As explained in Section *Graph Representation of Decomposed Environments*, a new graph representation of environment was constructed, where the graph nodes represent corners of the cells, and the edges represent cell coverage, contour-following, or inter-cell traversal. For a complete coverage, all cells including primary and transition cells are required to be covered by the robot at least once. This is equivalent to going through all cell coverage edges (transition cells) or edge pairs (primary cells) in the graph at least once. Since cell coverage edges or edge pairs are not connected, the robot should connect these coverage edges together using contour-following or inter-cell traversal edges. To find an efficient sequence of covering the cells, an optimization problem over the graph was formulated that targets to minimize the distance travelled by the robot for transitioning between the cells. Similar to the Rural Chinese Postman Problem (Pearn and Wu, 1995), the optimization problem would be an NP-complete combinatorial problem since the subset of required edges does not form a weakly-connected network.

In this work, the optimization problem was solved using a double DQN technique (Van Hasselt et al., 2015). As represented in **Algorithm 1**, double DQN trains a deep *Q* network $\left(Q_{\theta }\right)$ to approximate the action values through a DRL process. The reason that a DRL has been chosen over RL techniques is that the state space for coverage problem is generally large, and consequently there would be a large number of state-action pairs. Therefore, a function (deep network) would be required to approximate the action values (*Q* values). Two identical deep networks, online Q network $\left(Q_{\theta }\right)$ and target Q network $\left(Q_{\theta^{\prime }}\right)$, are utilized in the double DQN learning process. The purpose of using the target *Q* network is to reduce *Q*-values overestimation (Van Hasselt et al., 2015). Through this DRL-based technique, the robot interacts with the environment defined based on the proposed graph and learns how to choose the starting corner of the next cell in the coverage sequence. A pseudocode is provided in Table 1, which illustrates how the environment in DRL framework has been constructed based on the proposed graph information. When $Q_{\theta }$ is trained over the graph environment, the efficient coverage sequence of the cells would be equivalent to the optimal policy found by double DQN. The optimal action that the robot takes at each step of the coverage process is computed by Eq. 1.

$$a_{t}^{*}=\underset{a}{\arg\max}Q_{\theta }\left(S_{t},a\right),$$ (1)

where arepresents all possible actions that the robot can take at state $S_{t}$, which are the integer numbers in the set: [0, number of nodes), and $a_{t}^{*}$ is the optimal action taken by the agent at each state $S_{t}$, which is the largest *Q*-value approximated by $Q_{\theta }$. It is an integer number in the set: [0, number of nodes) that represents the starting corner of the next cell in the optimal coverage sequence.

**Algorithm 1.** Double DQN (Van Hasselt et al., 2015).

Initialize online network $Q_{\theta }$, target network $Q_{\theta^{\prime }}$, experience replay memory D **repeat** **for** each environment step: Select some action $a_{t}$ at state $S_{t}$ Execute $a_{t}$ and observe next state $S_{t+1}$ and reward $r_{t}$ Add $\left(S_{t},a_{t},S_{t+1},r_{t}\right)$ to D **for** each online network update step: Sample a random mini-batch of $\left(S_{t},a_{t},S_{t+1},r_{t}\right)$ from D Compute the target: $Y_{t}^{Q}=r_{t}+\gamma Q_{\theta }(S_{t+1},\underset{a}{\arg\max}Q_{\theta^{\prime }}\left(S_{t+1},a\right))$ Update the online network weight θ based on the error ${\left(Q_{\theta }\left(S_{t+1},a_{t}\right)-Y_{t}^{Q}\right)}^{2}$ **for** each target network update step: Update the target network weight: $\theta^{\prime }=\theta \;$

**TABLE 1:** Pseudocode for the python class *graphEnvironment* which includes details on how the methods for episode reset, transition and reward model, and creating the observation image are coded. The environment in DRL framework is constructed based on the proposed graph information.

**class** graphEnvironment:
** def** __init__(graph, start_corner, end_corner, max_steps):
** **episode_step ← 0
** **reward ← 0
** **done ← False
** **SIZE ← ceil ( sqrt (graph.num_corners))
** **episode_actions ← empty array
** **current_corner ← start_corner
** **state ← a list of 0s and 1s representing the robot current corner and already covered cells
** **final_state ← a list of 0s and 1s representing the robot end corner and required cells to be covered
** **final_reward ← 1
** **double_coverage_reward ← - 0.5
** **primary_cell_coverage_reward ← 0.05
** **transition_cell_coverage_reward ← 0.005
** **cost_scale ← 0.1
** **coverage_overlap_scale = 2
** def** reset():
** **episode_step ← 0
** **reward ← 0
** **done ← False
** **start_corner ← a random integer number in the set [0, graph.num_corners)
** **end_corner ← a random integer number in the set [0, graph.num_corners)
** **current_corner ← start_corner
** **state ← a list of 0s and 1s representing the robot current corner and a random set of already covered cells
** **final_state ← a list of 0s and 1s representing the robot end corner and a random set of required cells to be covered
** **episode_actions ← empty array
** **observation ← get_image()
** return** observation
** def** step(action):
** **episode_step ← episode_step + 1
** if** action belongs to a cell that has not been covered:
** **action_sequence ← graph.actionSequence (current_corner, action)
** **episode_actions ← concatenate (episode_actions, actionSequence)
** **reward ← 0
** for** act in action_sequence:
** **new_pos ← graph.nextCorner (current_corner, act)
** **cost ← graph.transitionCost (current_corner, act)/max_cost×cost_scale
** **reward ← reward - cost
** if** current_corner is in a primary cell:
** if** act is equivalent to cell coverage:
** **state ← update state by adding the current cell to the list of covered cells
** **reward ← reward + primary_cell_coverage_reward
** if** act is equivalent to contour-following and cell has already been covered or the contour-following is parallel to the coverage direction:
** **reward ← reward - cost×coverage_overlap_scale
** else:**
** if** act is equivalent to cell coverage:
** if** action != current_corner:
** **reward ← reward - cost×coverage_overlap_scale
** else**:
** **state ← update state by adding the current cell to the list of covered cells
** **reward ← reward + transition_cell_coverage_reward
** **current_corner ← new_pos
** **state ← update the robot current corner
** else**:
** **done ← False
** if** state==final_state or episode_step >= max_steps:
** **done ← True
** **new_observation ← get_image()
** **reward ← double_coverage_reward
** return** new_observation, reward, done
** if** all required cells have been covered:
** **final_path_cost ← graph.transitionCost (current_corner, end_corner)/max_cost×cost_scale ×(coverage_overlap_scale+1)
** **reward ← reward + final_reward - final_path_cost
** **action_sequence ← graph.actionSequence (current_corner, end_corner)
** **episode_actions ← concatenate (episode_actions, actionSequence)
** **current_corner ← end corner
** **done ← False
** if** state==final_state or episode_step >= max_steps:
** **done ← True
** **new_observation ← get_image()
** return** new_observation, reward, done
** def** get_image():
** **env ← 3-dimensional array with size of (SIZE, SIZE, 3) # equivalent to an image with 3 channels and width×height of SIZE×SIZE
** **env ← update the first channel to indicate the robot current corner
** **env ← update the second channel to indicate the already covered cells
** **env ← update the third channel to indicate the robot end corner
** **img ← convert env to an RGB image
** return** img

In the following, the DRL framework in this context is explained. More details on the definitions and steps are provided in the pseudocode of Table 1.

**Episodes** of the learning process start with a robot at a random node (corner) and a list of already covered cells which is created randomly. The state changes through interactions of the agent and the environment, which is defined based on our proposed graph representation of the environment. An episode ends when the agent reaches the goal (final) state. The agent is at the goal state when all the cell coverage edges are covered, and the robot is at the end node (corner) of the episode, which is also selected randomly. If the episode is not converging to the goal state, it will be forced to stop when the number of steps reaches a pre-defined maximum number of steps.

**State** includes 1) the robot’s current node (corner), 2) the list of already covered cells, and 3) the episode’s end node (corner). Since the model considered for the Q networks is a convolutional neural network, the state, S, at each step of the episode is a three-channel image created based on the observation made on the above three parameters, where the first channel has a nonzero value in the pixel corresponding to the robot’s current node number, the second channel has nonzero values in the pixels corresponding to the already covered cell numbers, and the third channel has a nonzero value in the pixel corresponding to the robot’s end node number. The size of this square image is calculated based on the total number of nodes.

**Action**, a, is an integer number in the set: [0, number of nodes) that represents a node number. Having access to its current state, the agent selects a node number from which the robot should start covering the next cell. There are four options (nodes) for starting coverage from at each primary cell and two options (nodes) for starting coverage from at each transition cell.

**Transition** is the process of applying the selected action at the current state and updating the state after observing the changes in the environment. If the selected action by agent is a node in an uncovered cell, the robot takes the shortest path from the current node to the selected node (as its action) over the graph. When the robot arrives at the selected node (as its action), it starts covering the cell and ends at another corner of the cell. This covered cell is added to the list of already covered cells in the new state. The robot’s current node (corner) is updated in the new state as well. If the selected action by agent is a node in an already covered cell, the state does not change, and a negative reward is assigned to that state-action pair.

**Rewards** are defined in a way that they would encourage the robot to take an efficient path for traversing all cell coverage edges in the proposed graph. The reward function is defined in a way that it would reward the agent for reaching the goal state with minimum travelled distance. The following is a description of the reward function. The pseudocode of Table 1 provides more detail on how these rewards are applied at each step of an episode.

- If the agent at a particular state selects a node to travel to (as its action) which belongs to an already covered cell, meaning that it decides to cover a cell that has already been covered before, the state (robot’s current node and the list of already covered cells) will not change and a large negative *double-coverage* reward of $R_{dc}=-0.5$ will be assigned to that state-action pair. $R_{dc}$ has a large negative value since covering an already covered cell (area) of the environment results in a large amount of unnecessary cost (extra travelled distance) while not helping the coverage task completion at all. The magnitude of $R_{dc}$ has been chosen to be much larger than the magnitude of the cost of transition ($C_{T}$ in Eq. 2) for travelling from current node to a node in an adjacent uncovered cell. This encourages the agent to avoid covering a cell more than once and try covering the uncovered cells in the environment instead. The exact value of $R_{dc}$ has been found by tuning the DRL hyperparameters over several sample indoor environments based on speed of convergence*, and not getting stuck in local minima.*

- If the agent at a particular state selects a node to travel to (as its action) which belongs to an uncovered cell, the transition model has two parts: 1) transition part which constitutes travelling from the current node to the selected node (as an action) using contour-following and inter-cell traversal edges, and 2) coverage part which constitutes covering the destination cell starting from the selected node (as an action). Therefore, the *coverage* reward assigned to the state-action pair, $R_{c}$, has two parts that encourages the agent to select nodes in the uncovered cells while minimizing the travelled distance for transition to the selected node.

$$R_{c}\;=\;C_{T}\;+\;R_{cc}.$$ (2)

Cost of transition, $C_{T}$, and *cell-coverage* reward, $R_{cc}$, are described below:

1) Cost of transition $\left(C_{T}\right)\;$ is a negative reward assigned for the cost of travelling from current node to the selected node (as an action). The total cost of transition is the summation of cost of traversing all edges in the graph path from current node to the selected node:

$$C_{T}=\sum_{i}C_{T_{i}}.$$ (3)

The cost of travelling from one node to an adjacent node in the graph has already been calculated offline and is included in the graph edge weights. The graph edge weights, $W_{T_{i}}$, are obtained based on the shortest distances between adjacent nodes of the graph. A primary cell coverage edge weight is zero since there is no extra travelled distance for covering a single primary cell. To find $C_{T_{i}}$ associated to every two nodes, the edge weight $\left(W_{T_{i}}\right)$, is normalized and then scaled based on a trial and error process while tuning the DRL framework rewards definition.

$$C_{T_{i}}=-0.1\left(\frac{W_{T_{i}}}{W_{T_{\max}}}\right)\;,$$ (4)

where $W_{T_{\max}}$ is the maximum edge weight in the entire graph.

2) A positive *cell-coverage* reward of $R_{cc}=0.05$ for covering an uncovered primary cell or a positive cell coverage reward of $R_{cc}=0.05\times 0.1=0.005$ for covering an uncovered transition cell is assigned to the coverage part. This positive $R_{cc}$ reward has been utilized to allow the agent learn if it is gradually getting closer to the goal state. The magnitude of $R_{cc}$ is in the order of the magnitude of the cost of transition ($C_{T}$ in Eq. 2) for travelling from current node to another node in an uncovered cell. This helps the agent to learn how to select the next cell in a way that the total episode reward is maximized instead of selecting the closest uncovered cell. $R_{cc}$ for covering an uncovered transition cell is smaller (by a factor of 10) than the corresponding parameter for a primary cell since the transition cells are always smaller than the primary cells. The exact values of $R_{cc}$ for both primary and transition cells have been found by trial and error through implementing the DRL optimization over several sample indoor environments. Improper values of $R_{cc}$ would increase the optimization convergence time and/or even lead to getting stuck in local minima.

- When the transition path includes a contour-following, the edge transition cost ($C_{T_{i}}$) is calculated similar to that in Eq. 4 as if the contour-following happens in an uncovered cell, and that it is not parallel to the cell coverage direction. However, if the contour-following happens in a covered cell or the contour-following path is parallel to the cell coverage direction, the cost would increase since coverage overlaps would be inevitable in those parts. For those cases, the transition cost is multiplied by a factor of 3 (obtained based on trial and error) to penalize the agent for the coverage overlaps (extra travelled distances) as it is indicated in Eq. 5.

$$C_{T_{i}}=-0.1\left(3\right)\left(\frac{W_{T_{i}}}{W_{T_{\max}}}\right).$$ (5)

- The magnitude of this transition cost should not be much larger than the magnitude of transition cost in Eq. 4 since the agent should still have a chance to take a contour-following path in a covered cell if it leads to a maximized total episode reward at the end.

- If applying an action at the current state results in reaching the goal state, where all the cells are covered and the robot is at the end corner of the episode, a large positive *final* reward of $R_{f}=1$ will be assigned to the current state-action pair. This large positive reward encourages the agent to continue taking transition paths and covering the cells to reach the goal state.

This double DQN approach, like many other Deep learning techniques, is slow at the training step since a large number of possible interactions with the environment needs to be fed to the learning networks. However, once the agent is trained on the environment, the execution speed would be fast. The agent would be capable of generating an efficient coverage path for any arbitrary start and/or end positions immediately (i.e., via a transfer learning). The user can determine which cells need to be covered (which could be equivalent to selecting the rooms that need to be disinfected), and the agent will generate the coverage path using the already trained model without wasting time to solve the problem for the new setting. The training step used in the DRL can be done offline on a more powerful processor and then the trained network can be uploaded onto the robot onboard hardware. At the execution step, the required computational power is not expensive, therefore, most of the commonly-used processors on the mobile robots will be able to run it in real time.

Similar to some other CPP works cited in the literature (e.g., Hameed et al., 2013; Xu et al., 2014; Lewis et al., 2017; Chen et al., 2019), our proposed technique considers a global path planning that finds an efficient sequence for covering different regions (cells) of the static and known environment, while minimizing the travelled distance (overlap) in the coverage path. Therefore, we assume that the hospital floor plans would be static and known in advance, and the DRL-based coverage planning can be carried out offline. The proposed Double DQN optimization technique is applicable for hospital disinfection since it is fast enough at the execution phase and suitable for transfer learning (i.e., it can manage new start/end corners, arbitrary areas of the environment selected for disinfection). The environment uncertainties such as unknown obstacles, dynamic objects, etc. and the robot constraints are usually considered in a local path planning algorithm, which needs to constantly acquire sensory information on the environment. Development of a local path planning method constitutes our future work through which we will compute a collision-free trajectory in real time based on the coverage path generated by our proposed global CPP technique.

## Results and Discussion

The proposed graph can be automatically created for any environment based on the steps described in Section *Environment Decomposition* and Section *Graph Representation of Decomposed Environments*. The environment data including the planar map of the environment is passed as input to a MATLAB script written by our team. The proposed algorithm creates and saves a file which contains all the information about the graph representation of the environment including nodes, edges, and transition costs and action sequences of travelling from one node to another. The time required for this process is in the order of seconds (using an OMEN HP laptop running Windows 10 with an Intel Core i7-9750H CPU @ 2.60 GHZ and 16 GB of RAM). The constructed graph is passed to a Python code, where the environment of the DRL framework has been created based on the information of the graph. The pseudocode for the Python class of the environment constructed based on the proposed graph is provided in Table 1. The Double DQN agent provided in the GitHub repository of keras-rl package has been utilized to create the Keras model and run the reinforcement learning episodes. The sequential model initialized for both online and target Q networks includes three convolutional layers followed by two dense layers. An epsilon-greedy policy and an Adam optimizer with learning rate of 25e-5 are considered for the training step.

**FIGURE 11:**
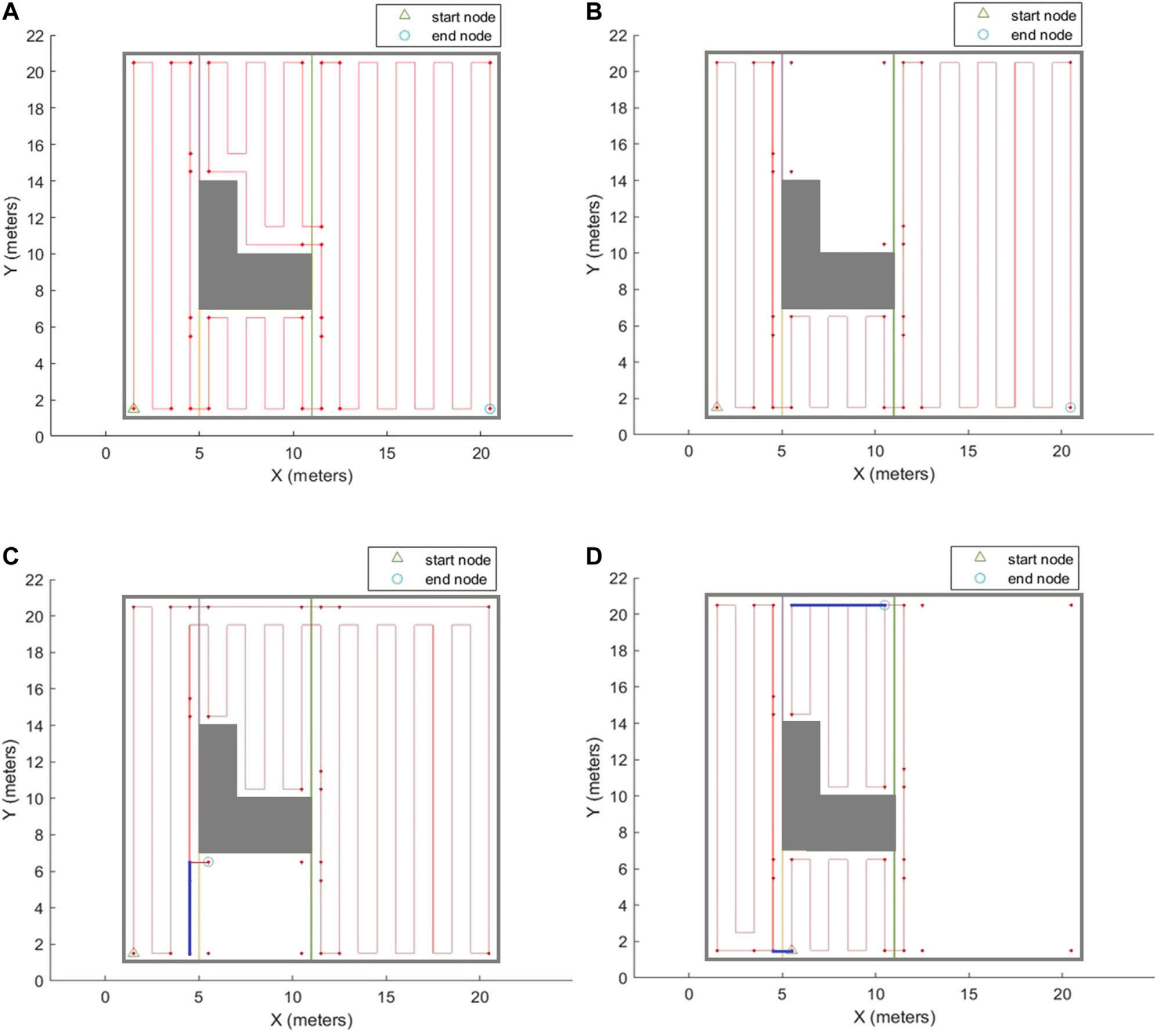
The complete coverage path generated by the proposed CPP technique based on the graph representation of environment. **(A)** Whole free space has been covered. **(B)** Cell 3 has been excluded from the area of interest. **(C)** Cell 2 has been excluded from the area of interest, and end corner position has changed. **(D)** Cell 4 has been excluded from the area of interest, and both start and end corner positions have changed. The blue lines represent the overlapped parts of the path.

After the agent is trained over the graph environment, it would be able to generate an efficient traversal sequence over the graph for different configurations of the input environment. Different configurations can be created by selecting different: 1) start and/or end positions for disinfection task, and/or 2) sets of the environment cells that need to be covered. In order to evaluate the performance of the proposed CPP technique, the coverage path has been generated in several environments. The environment shown in Figure 2, with modified cell decomposition shown in Figure 9, and the graph representation shown in Figure 10, has been chosen to demonstrate the performance of our proposed method. This simple environment has been chosen as the testbench evaluation environment since the optimal coverage path for different configurations of this environment is manually attainable. In the first scenario, the entire free space is required to be covered. The coverage planning is set to start from cell one and to end at cell four. Figure 11A shows the calculated coverage path. As it can be seen, there are no overlaps (extra travelled distance). The robot performs a contour-following motion in cell three to adjust the cell coverage start corner. The disinfection coverage diameter (W) in this scenario was set at 1 m. It should be noted that all cell coverage paths are assumed to be in vertical direction (parallel to the Y-axis).

As it was mentioned in Section *Coverage Optimization Over the Proposed Graph*, the coverage path planner can generate paths for different configurations of the environment which it has been trained for. It means that users can select any arbitrary area of interest in the environment to be covered (Figure 11B). They can also choose any arbitrary combination of the start and end corners for the disinfection task (Figures 11C,D). As it can be seen in Figure 11, the generated paths for different configurations of the environment are optimal, i.e., the extra travelled distance (overlap) is minimal.

**FIGURE 12:**
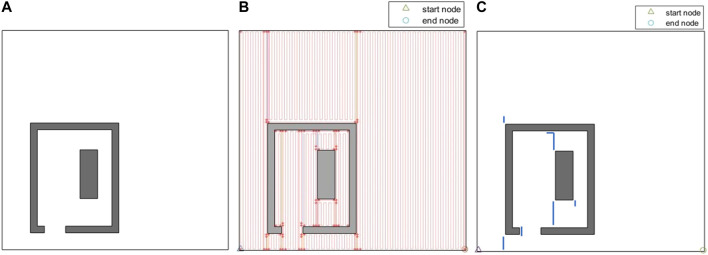
**(A)** An indoor environment with a single room and a single obstacle inside the room. **(B)** The complete coverage path generated by the CPP technique based on the graph representation of environment. The path stripes are shown by solid red lines. **(C)** The extra travelled distances (overlaps) in the generated coverage path. The overlapping parts of the path are shown by solid blue lines.

**FIGURE 13:**
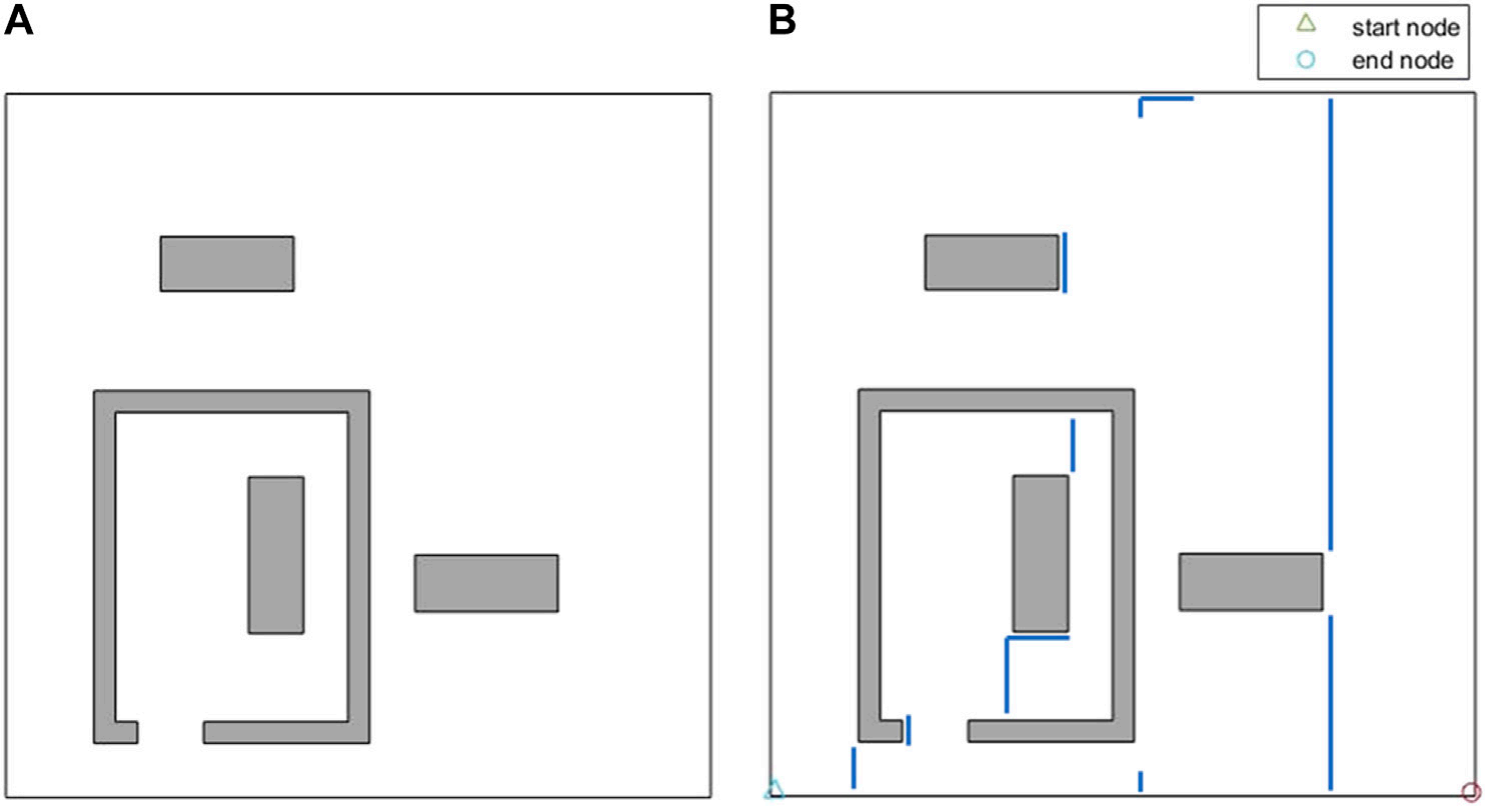
**(A)** An indoor environment including a single room with a single obstacle inside and two obstacles outside. **(B)** The extra travelled distances (overlaps) in the generated coverage. The overlapping parts of the path are shown by solid blue lines.

Figure 12A represents another indoor environment that comprises of a single room and a single obstacle. The generated path is shown in Figure 12B. Since the environment is larger than the simple environment in Figure 11, and that there is a higher number of path stripes over the environment, Figure 12C has been also generated to show the extra travelled distances (overlaps) in the generated coverage path. Figure 13A shows another environment that comprises of the same room, but two obstacles are added to the environment. The extra travelled distances (overlaps) in the generated path are shown in Figure 13B. The extra travelled distance (overlap) is the optimization metric used in the CPP problem. If two different CPP approaches generate two different complete coverage paths with no overlaps, the travelled distances of those two approaches will be equal, and the extra travelled distance will be zero in both cases. As the coverage overlap increases, the extra travelled distance increases. Therefore, the extra travelled distance (overlap) can be considered as the evaluation metric in the CPP problem. The complete coverage paths generated for both cases in Figures 12, 13 environments are quite efficient in terms of extra travelled distance since the ratios of their extra travelled distance to their total travelled distance are quite low (less than 2%).

**FIGURE 14:**
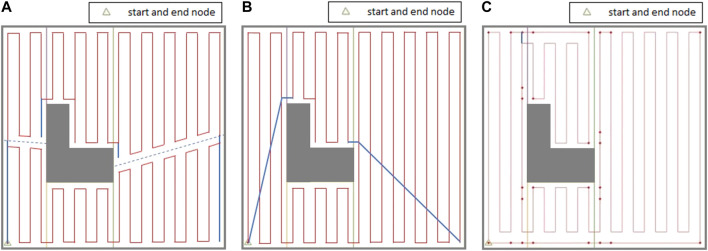
The complete coverage path generated by: **(A)** the CPP technique proposed in (Xu et al., 2014), **(B)** the CPP technique proposed in (Tung and Liu, 2019), and **(C)** the proposed CPP technique based on the graph representation of environment. The solid red lines represent the generated path, and the solid blue lines represent the overlapped parts of the path. The dashed blue lines show the common boundaries of the divided cells.

In the next step of the evaluation, the performance of the proposed CPP technique based on our proposed graph representation of environment has been compared with two well-known state-of-the-art CPP techniques suggested in the literature over two indoor environments. The CPP technique proposed in (Mannadiar and Rekleitis, 2010; Xu et al., 2011; Xu et al., 2014) duplicated selected edges of the Reeb Graph to make it Eulerian, and then solved a Chinese Postman Problem over the graph to find the efficient sequence of cell coverage with minimal travelled distance. Figure 14A shows the coverage path generated by the CPP technique proposed in (Xu et al., 2014) on the simple environment shown in Figure 2. As it can be seen in Figure 14A, there are many turns in the middle of cell one and cell four that happen because of splitting these two cells into two parts (dashed blue lines). The extra travelled distances (overlaps) are represented by solid blue lines. The generated coverage path is not optimal over this simple environment since the technique does not consider the start and end points of coverage for each cell. In their CPP technique, the robot has to return to the starting cell at the end of the coverage (Figure 14A).

The CPP technique proposed in many of the previous attempts (e.g., Jimenez et al., 2007; Hameed et al., 2013; Tung and Liu, 2019) utilized Genetic Algorithm (GA) optimization to find an efficient coverage sequence over the adjacency graph of the environment that visits each node (cell) at least once by solving a Traveling Salesman Problem. The coverage path generated by the CPP technique proposed in (Tung and Liu, 2019) is shown in Figure 14B. As it can be seen in Figure 14B, the robot needs to traverse through the middle of the cells one and four to get back to the starting point which results in extra travelled distances (overlaps) represented by solid blue lines in cells one and four. In their CPP technique, the robot has to return to the starting point at the end of the coverage.

A coverage path has been also generated using our proposed method on the same environment with the exact same start and end position. Results are given in Figure 14C. Our proposed method outperforms the CPP techniques in (Xu et al., 2014) and (Tung and Liu, 2019) in terms of total travelled distance. The extra travelled distance in the path generated by our approach is about 5% and 3.6% of the extra travelled distance resulted by applying the CPP technique in (Xu et al., 2014) and (Tung and Liu, 2019), respectively. The robot takes contour-following paths to adjust its position for starting the cell coverage at each cell. After arriving at the cell coverage start point found through the optimization, the robot covers the cell with no overlaps with the previously covered part of the cell contour. This can be seen in all the cells in Figure 14C. The number of turns in the path generated by our approach is about 65% of the number of turns resulted by applying the CPP technique in (Xu et al., 2014) since the robot does not need to split the cells to get back to the starting cell, and it takes contour following paths instead. The technique proposed in (Tung and Liu, 2019) produced similar number of turns to that in our proposed technique. A comparison of the extra travelled distance and number of turns is shown in Table 2. In Table 2, the extra travelled distance ratio is defined as the extra travelled distance using our proposed technique divided by the extra travelled distance utilizing other techniques in (Xu et al., 2014; Tung and Liu, 2019). Similarly, the number of turns ratio is defined as the number of turns produced through the use of our proposed technique divided by the number of those from other techniques in (Xu et al., 2014; Tung and Liu, 2019). The smaller extra travelled distance ratio or number of turns ratio are in Table 2, the better our technique has worked in comparison with other techniques.

**TABLE 2:** A comparison of the extra travelled distance and number of turns resulted in the proposed CPP technique based on the graph representation of environment and CPP techniques proposed in (Xu et al., 2014) and (Tung and Liu, 2019).

CPP technique proposed in other works	CPP technique proposed in (Xu et al., 2014)		CPP technique proposed in (Tung and Liu, 2019)	
Environment	Environment of Figure 14	Environment of Figure 15	Environment of Figure 14	Environment of Figure 15
Extra travelled distance ratio ×100	5%	42%	3.6%	43%
Number of turns ratio ×100	65%	45%	≈100%	≈100%

**FIGURE 15:**
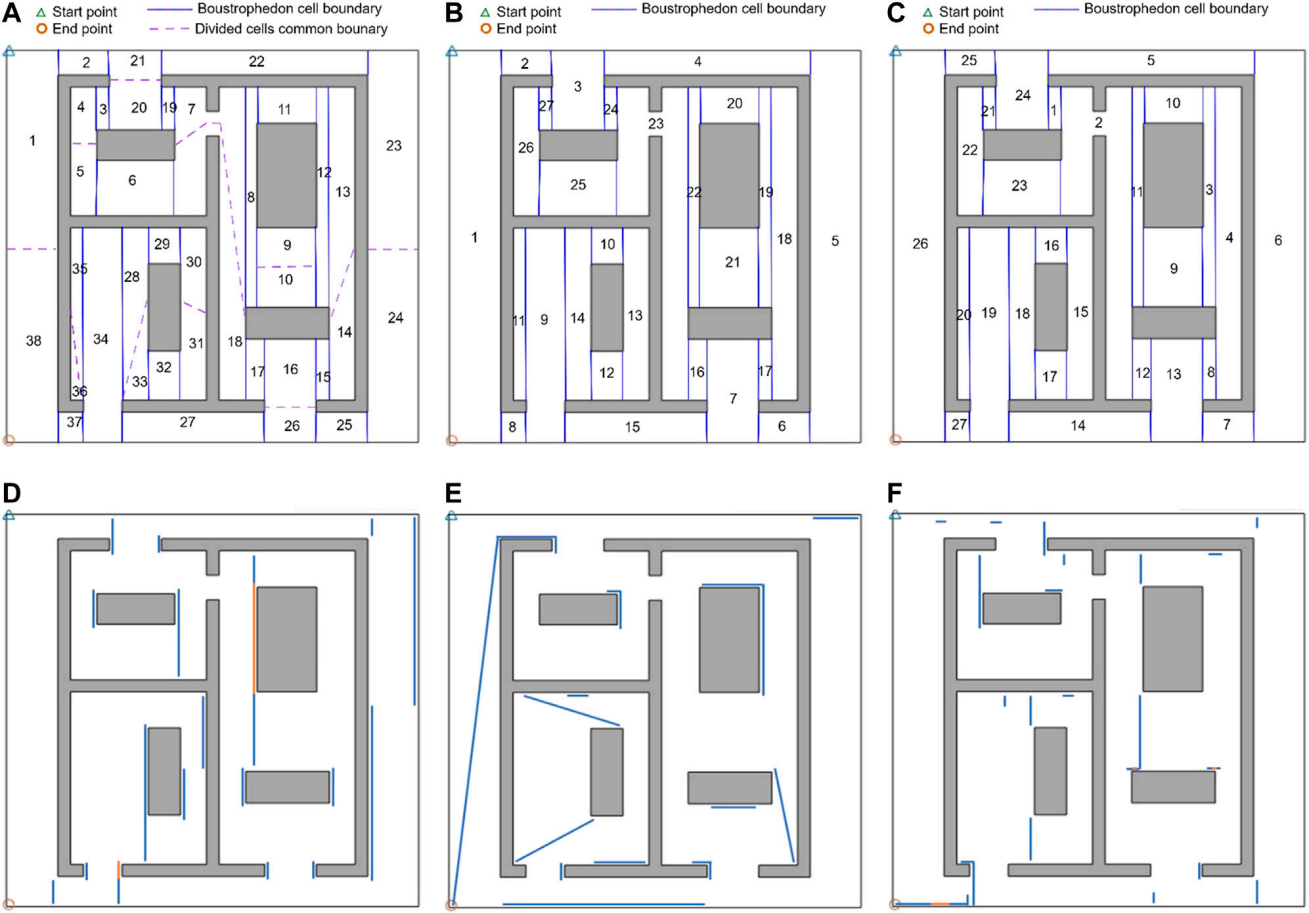
The cell coverage sequence by: **(A)** the CPP technique proposed in (Xu et al., 2014), **(B)** the CPP technique proposed in (Tung and Liu, 2019), and **(C)** the proposed CPP technique based on the graph representation of environment The extra travelled distances (overlaps) in the generated coverage path by: **(D)** the CPP technique given in (Xu et al., 2014), **(E)** the CPP technique proposed in (Tung and Liu, 2019), and **(F)** the proposed CPP technique based on the graph representation of environment. The overlapping parts of the path are shown by solid blue lines. The solid orange lines.

The proposed CPP technique based on the graph representation of environment and other techniques (Xu et al., 2014; Tung and Liu, 2019) have been applied to generate a complete coverage path on a more complicated indoor environment, as shown in Figure 15. The sequences of covering the cells using the proposed techniques in (Xu et al., 2014) and (Tung and Liu, 2019) are shown in Figures 15A,B respectively. The extra travelled distances (overlaps) in the path generated by the other techniques in (Xu et al., 2014) and (Tung and Liu, 2019) are depicted in Figures 15D,E, respectively.

The proposed CPP technique based on the graph representation of the environment has been applied over the same environment with same start and end points. The sequence of covering the cells and the extra travelled distance (overlaps) in the path generated by our proposed method are shown in Figures 15C,F respectively. A comparison of the extra travelled distance and number of turns has been shown in Table 2. As it can bee seen in Figure 15F, the extra travelled distance (overlap) has been decreased in the generated coverage path by our proposed method. The extra travelled distance in the path generated by our proposed approach is about 42% and 43% of the extra travelled distance resulted by applying the other CPP technique in (Xu et al., 2014) and (Tung and Liu, 2019), respectively. This cost reduction is a result of utilizing the proposed graph representation of the environment, which was created considering three modifications described in Section *Graph Representation of Decomposed Environments*. This improvement can decrease the disinfection cost dramatically since the hospital disinfection is a repetitive task which should be done based on certain routines. The decrease in the cost is more significant when our proposed method is applied over larger indoor environments like hospitals.

In addition to the extra travelled distance (overlap), the number of turns in the path generated for environment of Figure 15 is 45% of the total number of turns resulted by applying the CPP technique in (Xu et al., 2014). The number of turns is decreased because the proposed graph representation of the environment makes it possible for the robot to take a contour-following edge for adjusting each cell coverage start point or crossing a cell. The CPP technique in (Xu et al., 2014) split the duplicated edges, so the robot performed extra turns at each side of the splitting edges. This decrease in the number of turns decreases the disinfection task completion time and cost. The number of turns in the path generated by applying our technique is similar to the number of turns produced by applying the technique proposed in (Tung and Liu, 2019).

At the execution step, the required computational power was not expensive. Therefore, most of the commonly-used processors on the mobile robots will be able to run it in real time. For example, using an OMEN HP laptop running Windows 10 with an Intel Core i7-9750H CPU @ 2.60 GHZ and 16 GB of RAM, the execution of the algorithm for the environment of Figure 15 took about 30 s.

## Conclusion

Mobile robots make the hospital disinfection process safer and more effective. Central to the autonomous disinfection, a CPP technique was presented in this paper which decreases the time and cost for robotic disinfection of hospitals. The proposed technique utilizes a new graph representation of the environment. This graph representation of the environment is created offline considering a more flexible version of Boustrophedon cell decomposition method, taking both contour-following paths in cells, and the corners of cells as start and end points of cell coverage into account. The efficient cell coverage sequence is found by solving an optimization problem over the graph using a double DQN technique. The generated coverage path by the proposed technique has been compared with those generated by two state-of-the-art CPP approaches over two indoor environments. The results indicate that the travelled distance and number of turns are reduced when using our proposed method. In particular, the extra travelled distance in the path generated by the proposed approach was in the range of 3.6% to 43% of the extra travelled distance resulted from applying other CPP techniques cited in the literature, (Xu et al., 2014; Tung and Liu, 2019), depending on the complexity of the environment. Furthermore, the number of turns was 45% to 65% of the total number of turns resulted when applying one of the CPP techniques cited in the literature, (Xu et al., 2014). This will lead to an improved completion time and cost for disinfecting hospitals using unmanned systems.

The learning time in the double DQN is in the order of hours which indicates that the agent should be trained offline. However, once the agent is trained over an environment, the in-field execution time will be in the order of seconds (with an Intel Core i7-9750H CPU @ 2.60 GHZ and 16 GB of RAM). Additionally, the trained model by double DQN technique is robust to changes in the start and/or end states of the robot used for the disinfecting task. It is also robust to excluding some cells from the disinfection target area, so regions of interest to disinfect can be prioritized on the fly. For future works, we will apply other DRL techniques over our proposed graph to decrease the training time. To further our research, we intend to extend this work to scenarios, where multiple disinfecting robots are employed for doing the task collectively. This will decrease the total operation time significantly due to the division of workload over all robots, which can be incorporated to the current problem formulation under the DRL method. Also, further works needs to be done on adding constraints and uncertainties to the problem formulation, for instance, uncertainties in the obstacles position (including unknown static obstacles and dynamic obstacles), constraints and uncertainties in mobile robot motion, constraints on the battery capacity and access to the charging stations will be considered.

## Data Availability

The raw data supporting the conclusions of this article will be made available by the authors, without undue reservation.
